# Patulin Biodegradation by *Rhodosporidiobolus ruineniae* and *Meyerozyma guilliermondii* Isolated From Fruits

**DOI:** 10.1002/mbo3.70198

**Published:** 2026-01-06

**Authors:** Yidan Ji, Sung‐Yong Hong, Jinhuan Qu, Qing Chu, Shuxian Ma, Ae‐Son Om

**Affiliations:** ^1^ Department of Food and Nutrition Hanyang University Seoul Republic of Korea

**Keywords:** (E)‐ascladiol, biodegradation, *Meyerozyma guilliermondii*, patulin, *Rhodosporidiobolus ruineniae*, yeast

## Abstract

Patulin (PAT) is a toxic secondary metabolite produced by certain species of *Penicillium* and *Aspergillus* on pome fruits. In this study, we isolated *Rhodosporidiobolus ruineniae* (*R. ruineniae*) and *Meyerozyma guilliermondii* (*M. guilliermondii*) from a peach and an apple as candidates for PAT degradation, respectively, and investigated the effects of three key parameters (incubation time and temperature, and initial PAT concentration) on PAT removal rates, and the mechanism involved in PAT degradation by the yeast strains. The PAT degradation rate by the yeast strains was dependent on the three key parameters. Both yeast strains were able to degrade 1 μg mL^−1^ of PAT to below the regulatory limit (50 µg L^−1^) at 60 h when they were incubated at 35°C. The PAT removal by the yeast strains was not due to either binding onto yeast cell walls or degradation by extracellular fractions of the yeast culture among three yeast cell fractions (cell walls, extracellular, or intracellular fractions). The use of spheroplast or intracellular enzymes confirmed that PAT degradation occurred inside the yeast cells. Moreover, the PAT degradation ability was inducible in *M. guilliermondii*. LC/MS/MS analysis showed that (E)‐ascladiol is the sole PAT biodegradation product from both yeast strains. Our data demonstrated that both yeast strains were able to degrade PAT and produce (E)‐ascladiol, a less toxic product. These results could be exploited for practical applications to efficiently control PAT on fruits such as apples and peaches.

## Graphical abstract

This study investigated the effects of incubation time and temperature, and initial patulin concentration on patulin removal rates, and the mechanism involved in patulin degradation by *Rhodosporidiobolus ruineniae* and *Meyerozyma guilliermondii*. The patulin degradation rate by the yeast strains was dependent on the three key parameters. Both yeast strains were able to degrade patulin and produce (E)‐ascladiol, a less toxic product.

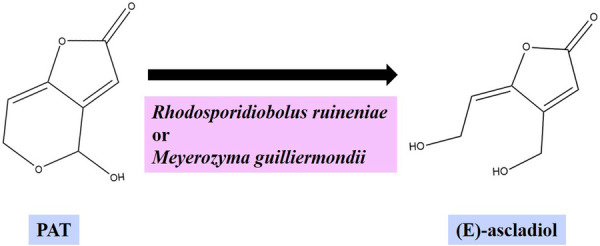

## 1 Introduction

Patulin (4‐hydroxy‐4H‐furo[3,2c]pyran‐2[6H]‐one, PAT) is a toxic secondary metabolite produced by various fungi such as *Penicillium* sp., *Aspergillus* sp., and *Byssochlamys* sp. on mainly fruits and vegetables, including apples, peaches, pears, and their products (Moake et al. 2005). PAT contamination occurs in rotten fruits and vegetables decayed by the postharvest pathogens when they are stored under relatively high moisture and moderate temperature for a long period of time. The PAT contamination of fruits and vegetables poses a serious health risk to consumers and is a major issue in food safety because it is classified as a Group 3 carcinogen by the International Agency for Research on Cancer (IARC) (Ianiri et al. 2017; Tannous et al. 2018). The PAT toxicity includes immunotoxic, neurotoxic, genotoxic, teratogenic, and carcinogenic effects as a chronic health risk, whereas it can cause edema, ulceration, inflammation, and gastrointestinal and alveolar hemorrhage as acute poisoning (Fu et al. 2021; Moake et al. 2005; Puel et al. 2010; Tannous et al. 2018). The underlying mechanism of PAT toxicity has been proposed due to adduct formation with thiol‐containing cellular components such as glutathione and cysteine‐containing proteins, leading to inhibition of protein and DNA syntheses and disruption of transcription and translation (Fliege and Metzler 1999, 2000; Moake et al. 2005). Thus, the Codex Alimentarius Commission (CAC), along with the US Food and Drug Administration (FDA) and European Commission (EC), has established a maximum tolerable limit (50 µg L^−1^) of PAT in apple‐based products (Codex Alimentarius Commission [CAC] 2003; European Commision [EC] 2023; US Food and Drug Administration [FDA] 2005). Thereafter, many regulatory agencies worldwide, such as Korea Ministry of Food and Drug Safety (MFDS), started to place limitations on the PAT content in single‐strength and reconstituted apple juice (Kang et al. 2010).

The PAT is also highly stable under acidic conditions and resistant to thermal denaturation, which makes it difficult to remove it from food products (Moake et al. 2005). Several strategies have been proposed for PAT detoxification, including physical methods such as adsorption by activated charcoal, chemical decontamination such as degradation by food‐grade additives, and biological control methods such as degradation by enzymes or microorganisms, and adsorption by cell walls (Moake et al. 2005; Wright 2015; Zheng et al. 2021). However, there may be some problems in the use of physical and chemical methods for PAT detoxification. Both methods can cause adverse effects on the organoleptic, physicochemical, and nutritional quality of food as well as limited efficacy and high cost (Kabak et al. 2006). Thus, the use of biological control methods is considered a powerful potential alternative method.

In recent years, several microorganisms, including some bacteria such as *Lactobacillus* sp. and *Gluconobacter* sp., and some yeasts such as *Saccharomyces* sp., *Pichia* sp., and *Candida* sp., and a filamentous fungus *Acremonium* sp. have shown PAT detoxification ability (Fu et al. 2021; Mita et al. 2023; Zheng et al. 2021). *Lactobacillus rhamnosus* and *Gluconobacter oxydans* were able to remove 70%–94% of PAT by binding onto their cell walls, whereas *Saccharomyces cerevisiae* (*S. cerevisiae*), *Meyerozyma caribbica* (*M. caribbica*; formerly *Pichia caribbica* [*P. caribbica*]), *Candida guilliermondii* (*C. guilliermondii*), *Kluyveromyces marxianus* (*K. marxianus*), *Acremonium* sp., *Cyberlindnera* sp., and *Wickerhamomyces* sp. were able to degrade 92%–100% of PAT by their enzymes (Chen et al. 2017; M. Li et al. 2018; J. Li et al. 2020; Markov et al. 2019; Mita et al. 2023; Song et al. 2024; Yang et al. 2024; Zhang et al. 2025; Zheng et al. 2016; Zhou et al. 2025). Many of the yeasts were isolated from soil and only a few were from food products such as apples. Since yeasts isolated from food products are considered favorable for application to PAT‐contaminated foods, in this study, we isolated two PAT‐degrading yeast strains, *Rhodosporidiobolus ruineniae* (*R. ruineniae*) and *Meyerozyma guilliermondii* (*M. guilliermondii*; formerly *Pichia guilliermondii*, anamorph *C. guilliermondii*), from a peach and an apple, respectively, and investigated the mechanism involved in PAT degradation and the major biodegradation product. Our results showed that PAT (1 μg mL^−1^) was completely degraded (100%) by each yeast strain at 72 h and 35°C, and that intracellular enzymes from the yeast strains produced (E)‐ascladiol as a PAT degradation product. This study reports conversion of PAT to (E)‐ascladiol, a less toxic product, by *R. ruineniae*.

## 2 Materials and Methods

### 2.1 Chemicals and Reagents

PAT standard (≥ 98.0% purity), HPLC grade acetic acid, Tween 80, proteinase K, lyticase, ethanol, chloramphenicol, tetracycline, and disodium ethylenediaminetetraacetic acid (Na_2_EDTA) were purchased from Sigma‐Aldrich Co. (St. Louis, MO, USA). Ethyl acetate and d‐sorbitol were obtained from Daejung Chemical Co. (Seoul, South Korea), whereas sodium carbonate (Na_2_CO_3_) and potassium acetate were from Samchun Chemical Co. (Seoul, South Korea). Tris base and sodium dodecyl sulfate (SDS) were obtained from Bio‐Rad (Hercules, CA, USA). Anhydrous sodium sulfate (Na_2_SO_4_), sodium acetate, and isopropanol were purchased from Junsei Chemical Co. (Tokyo, Japan), while acetonitrile (ACN, HPLC grade) was from Supelco (Darmstadt, Germany).

### 2.2 PAT Standard Solutions

A PAT stock solution (200 μg mL^−1^) was prepared by mixing 5 mg of PAT powder with 25 mL of ethyl acetate and stored at −20°C. Five levels of PAT standard solutions (0.1, 0.2, 0.5, 1.0, and 2.0 μg mL^−1^) were prepared freshly by dilutions of the stock solution with ethyl acetate. Then, 1 mL of each standard solution was evaporated to dryness under nitrogen at 60°C. The residue was dissolved in 1 mL of acidified sterile distilled water (DW; pH 4.0, adjusted with acetic acid), filtered through a 0.2 μm sterile syringe filter (Hyundai Micro Co. Seoul, Korea), and stored at −20°C until use.

### 2.3 Isolation of Yeasts From Peaches and Apples

Fresh peaches (cultivar Daewol) and apples (cultivar Aori) were collected from Yeongcheon and Yeongju, respectively, Gyeongsangbuk‐do in Korea and used to search for PAT‐degrading yeast strains. After microorganism suspensions were obtained from the surface of the peaches and apples by the cotton swab method using 0.85% sterile NaCl solution, they were inoculated onto potato dextrose agar (PDA; MB Cell, Seoul, Korea) plates containing 0.5 μg mL^−1^ of chloramphenicol and 0.5 μg mL^−1^ of tetracycline. The PDA plates were then incubated at 30°C for 3 days. Yeast colonies were isolated by inoculation onto yeast extract peptone dextrose (YPD; MB Cell, Seoul, Korea) agar plates, which were then incubated at 30°C for 3 days.

### 2.4 Identification of Isolated Yeasts

For yeast genomic DNA isolation, a small‐scale miniprep method was used according to a Cold Spring Harbor Laboratory course manual with minor modifications (Amberg et al. 2005). Briefly, yeast cells were cultured in 5 mL of YPD at 30°C with shaking at 150 rpm overnight. After centrifugation at 2000 rpm for 5 min, the yeast cells were resuspended in 0.5 mL of sorbitol solution containing 1 M sorbitol and 0.1 M Na_2_EDTA (pH 7.5). Then, 0.02 mL of lyticase (0.5 mg mL^−1^) was added to it and it was incubated at 37°C for 1 h. After centrifugation at 2000 rpm for 1 min, yeast cells were resuspended in 0.05 mL of 10% SDS plus 0.5 mL buffer solution containing 50 mM Tris·Cl (pH 7.4) and 20 mM Na_2_EDTA (pH 7.5), and it was incubated at 65°C for 30 min. Next, after 0.2 mL of 5 M potassium acetate was added to it, it was incubated on ice for 1 h. Finally, one volume of 100% isopropanol was added to the supernatant after centrifugation at 2000 rpm for 5 min, and it was incubated at room temperature for 5 min. After centrifugation for 5 min, the air‐dried DNA pellet was resuspended in 0.3 mL of TE (pH 7.4).

Yeast isolates were identified using DNA sequencing of the internal transcribed spacer 1 (ITS1)‐5.8S rDNA‐ITS2 region on yeast rDNA (White et al. 1990). Briefly, the ITS region of the isolated genomic DNA was amplified using two specific primers (ITS1 and 4) for identification of yeast species. The primer sequences are as follows: ITS1 (5′‐TCCGTAGGTGAACCTGCGG‐3′, forward) and ITS4 (5′‐TCCTCCGCTTATTGATATGC‐3′, reverse). Polymerase chain reaction (PCR) was performed at 95°C for 5 min, followed by 35 cycles of 95°C for 1 min (denaturation), 55°C for 1 min (annealing), and 72°C for 2 min (extension), and 72°C for 10 min (final extension). After the PCR products were separated on 1.2% (w/v) agarose gels by electrophoresis and purified using AccuPep PCR/Gel Purification Kit (Bioneer, Daejeon, Korea), they were sequenced by Biofact Co. (Daejeon, South Korea). Then, the yeast isolates were identified by the local similarity between DNA sequences of the PCR products and DNA sequences of yeast strains retrieved from GenBank in National Center for Biotechnology Information (NCBI) using BLASTn (Basic Local Alignment Search Tool nucleotide).

The phylogenetic tree was constructed using MEGAX program (v 11) based on the neighbor‐joining (NJ) method (Saitou and Nei 1987) along with DNA sequences of identified yeast species.

### 2.5 PAT Extraction and Determination of the Levels of PAT by HPLC

PAT was extracted from samples, which were used for measuring PAT recovery and removal rates, by the AOAC official method 995.10 with slight modifications as described previously (AOAC International 2000; X. Wei et al. 2022). Briefly, each sample was extracted twice with 5 mL of ethyl acetate and washed with 1 mL of 1.5% sodium carbonate solution. Then, it was dried over anhydrous sodium sulfate and evaporated to dryness under nitrogen at 60°C. The residue was dissolved in 1 mL of acidified sterile DW (pH 4, adjusted with acetic acid) and filtered through a 0.2 μm syringe filter (Hyundai Micro Co., Seoul, Korea) prior to injection onto a high‐performance liquid chromatography (HPLC).

A HPLC system (LC‐20AT; Shimadzu, Tokyo, Japan), which was equipped with a UV detector (UVD, SPD‐10A, Shimadzu, Tokyo, Japan), was used to detect and quantify PAT at 276 nm. Separation of analytes was carried out on a ZORBAX Eclips plus C18 column (4.6 mm × 250 mm, 5 μm particle size; Agilent, Santa Clara, CA, USA) with a flow rate of 0.5 mL min^−1^. The column oven temperature was set at 32°C, and the injection volume of the samples was 20 μL. The mobile phase was composed of 10% ACN (ACN: DW = 10:90, v/v), giving a total run time of 30 min.

The linearity of a series of PAT concentrations in the HPLC method was evaluated by a calibration curve using five levels of PAT standard solutions (0.1, 0.2, 0.5, 1, and 2 μg mL^−1^). The calibration curve of PAT was constructed by plotting the peak areas (*y*‐axis) versus PAT concentrations (*x*‐axis) in HPLC‐UVD analysis. The linearity was then determined by linear regression analysis and expressed as a coefficient of determination (*r* ^2^).

To determine the recovery rate of PAT, McIlvaine buffer (citric acid‐Na_2_HPO_4_, pH 4) solution was spiked with PAT standard solution (200 μg mL^−1^) to give concentrations of 0.2, 0.5, and 1 μg mL^−1^ PAT. PAT extraction from the spiked samples was performed by the procedures described above. After levels of PAT in the samples were analyzed by HPLC‐UVD, the recoveries were calculated by the following equation and expressed as mean ± standard deviation (SD):

$$\mathrm{Recovery}=\frac{\text{PAT concentration equivalent to the peak area measured from the spiked sample × }100}{\text{PAT concentration used for spiking the sample}}.$$

The repeatability (within‐day precision) was determined by three consecutive injections of PAT solutions, which were extracted from the spiked samples, within a day. The within‐day precision was expressed as relative standard deviation (*RSDr*) of PAT levels measured in triplicate.

The sensitivity of the HPLC‐UVD method was determined by a limit of detection (LOD) and limit of quantification (LOQ). They were calculated by the following equation using the slope (*S*) of the calibration curve, which was obtained from assessment of recovery rates, and the standard deviation (SD) of the response.

$$\mathrm{LOD}=\frac{3.3\times \mathrm{SD}}{S},$$

$$\mathrm{LOQ}=\frac{10\times \mathrm{SD}}{S}.$$

### 2.6 Screening for PAT Removal Yeasts

Each yeast strain was cultured at 28°C with shaking at 150 rpm for 48 h after inoculation of 100 μL of an overnight seed culture (OD_600nm_ = 0.01) into 5 mL YPD (pH 4) spiked with 1 μg mL^−1^ of PAT. Five milliliters of YPD (pH 4) spiked with 1 μg mL^−1^ of PAT, to which sterile DW (100 μL) was added, was used as a control. PAT was then extracted and analyzed by the procedure described above. PAT removal rates were calculated and expressed as mean ± standard deviation (SD).

### 2.7 PAT Removal Rates by *R. ruineniae* or *M. guilliermondii* Under Different Conditions

The effects of incubation time, incubation temperature, and initial PAT concentration on PAT removal rates by yeast strains were tested using McIlvaine buffer solution (pH 4). After each yeast strain was cultured in YPD at 28°C with shaking at 150 rpm overnight, it was washed with sterile DW twice and resuspended in a sterile McIlvaine buffer solution (pH 4). Then, to investigate the effect of incubation time, 100 μL of yeast cell suspension, which was adjusted to 1 × 10^9^ cells mL^−1^, was inoculated into 5 mL of sterile McIlvaine buffer solution (pH 4) containing 1 μg mL^−1^ of PAT, and it was incubated at 35°C with shaking at 150 rpm for 0, 6, 12, 24, 48, and 72 h. A sterile McIlvaine buffer solution (pH 4) containing only PAT without yeast was used as a control.

To examine the effect of incubation temperature, 5 mL of sterile McIlvaine buffer solution (pH 4) containing 1 μg mL^−1^ of PAT was incubated at 25°C, 30°C, 35°C, and 40°C with shaking at 150 rpm for 48 h after inoculation of 100 μL of yeast cell suspension (1 × 10^9^ cells mL^−1^). A sterile McIlvaine buffer solution (pH 4) containing only PAT without yeast was used as a control.

To analyze the effect of initial PAT concentration, 5 mL of sterile McIlvaine buffer solution (pH 4) was spiked with 0.2, 0.5, 1, 2, and 3 μg mL^−1^ of PAT. After inoculation of 100 μL of yeast cell suspension (1 × 10^9^ cells mL^−1^), it was incubated at 35°C with shaking at 150 rpm for 48 h. A sterile McIlvaine buffer solution (pH 4) containing only PAT without yeast was used as a control. After centrifugation at 4000 rpm for 10 min, PAT was extracted from supernatant and the amount of PAT was analyzed by HPLC‐UVD as described above.

### 2.8 Mechanism of PAT Removal by *R. ruineniae* or *M. guilliermondii*

As described above, 5 mL of sterile McIlvaine buffer solution (pH 4) containing 1 μg mL^−1^ of PAT was incubated at 35°C with shaking at 150 rpm for 48 h after inoculation of 100 μL of yeast cell suspension (1 × 10^9^ cells mL^−1^). Then, after centrifugation at 4000 rpm for 10 min, PAT was extracted from supernatant and the level of PAT was analyzed by the procedure described above. Then, in order to test whether PAT was adsorbed onto yeast cell wall, two different methods were used as follows: (1) after the yeast cell pellet was washed twice with sterile DW and resuspended in a sterile McIlvaine buffer solution (pH 4), the yeast cells were disrupted by ultrasonication using a Ultrasonic processor (GE 50, GE Healthcare, Chicago, IL, USA) for 30 min at a 40% amplitude and a 20 kHz frequency. Next, yeast cell walls were precipitated by centrifugation at 4000 rpm for 10 min and washed twice with sterile DW. After the yeast cell wall was resuspended in 5 mL of sterile McIlvaine buffer solution (pH 4), PAT was extracted from the yeast cell wall suspension and the amount of PAT was analyzed as described above; (2) after the yeast cell pellet was washed twice with sterile DW, the yeast cells were ground under liquid N_2_ and the disrupted yeast cells were resuspended in sterile McIlvaine buffer solution (pH 4). Then, after centrifugation at 4000 rpm for 10 min, the yeast cell wall fraction was washed twice with sterile DW. Next, after the yeast cell walls were resuspended in 5 mL of sterile McIlvaine buffer solution (pH 4), PAT was extracted from the yeast cell wall suspension and the amount of PAT was analyzed by HPLC‐UVD as described above.

For PAT removal test using heat‐treated cells (dead cells), yeast cell suspension (1 × 10^9^ cells mL^−1^) was autoclaved at 121°C for 15 min. Then, 5 mL of sterile McIlvaine buffer solution (pH 4) containing 1 μg mL^−1^ of PAT was incubated at 35°C with shaking at 150 rpm for 48 h after inoculation of 100 μL of dead yeast cell suspension. A sterile McIlvaine buffer solution (pH 4) containing only PAT without yeast was used as a control. After centrifugation at 4000 rpm for 10 min, PAT was extracted from supernatant and the level of PAT was determined by HPLC‐UVD as described above.

For PAT removal test using yeast cell‐free filtrate, the cell‐free filtrate was prepared as follows. After 100 μL of yeast cell suspension (1 × 10^9^ cells mL^−1^) was inoculated into 5 mL of YPD medium (pH 4) or sterile McIlvaine buffer solution (pH 4), they were incubated at 35°C with shaking at 150 rpm for 48 h. Then, after centrifugation at 4°C and 4000 rpm for 10 min, 100 μL of supernatant from each culture was added to 5 mL of new YPD medium (pH 4) or sterile McIlvaine buffer solution (pH 4) containing 1 μg mL^−1^ of PAT, and they were incubated at 35°C with shaking at 150 rpm for 48 h. YPD medium (pH 4) and McIlvaine buffer solution (pH 4) without the yeast culture, which were spiked with 1 μg mL^−^^1^ of PAT, were used as controls after addition of YPD or McIlvaine buffer solution (100 μL). Finally, PAT was extracted from the samples and the amount of PAT was analyzed as described above.

For PAT removal test using yeast spheroplast, the spheroplast was prepared by treatment of yeast cell suspension containing 1 M sorbitol with lyticase (0.5 mg mL^−^^1^) at 37°C for 1 h. The spheroplast pellet was resuspended in 5 mL of sterile McIlvaine buffer solution (pH 4) containing 1 μg mL^−1^ of PAT after centrifugation at 4°C and 4000 rpm for 10 min. Then, the buffer solution (pH 4) containing 1 μg mL^−1^ of PAT was incubated at 35°C with shaking at 150 rpm for 48 h after inoculation of 100 μL of spheroplast suspension (1 × 10^9^ cells mL^−1^). After centrifugation at 4000 rpm for 10 min, PAT was extracted from supernatant and the level of PAT was determined by HPLC‐UVD as described above. Also, for identification of PAT degradation products, 5 mL of sterile McIlvaine buffer solution (pH 4) containing 5 μg mL^−1^ of PAT was used instead of the use of 1 μg mL^−1^ of PAT, and it was incubated at 35°C with shaking at 150 rpm for 0, 12, and 24 h after inoculation of the spheroplast suspension.

For PAT removal test using intracellular enzymes from yeast cells, the yeast cells, which had been cultured in YPD, were ground under liquid N_2_ as described above for isolation of yeast cell wall fractions. Then, after centrifugation at 4°C and 4000 rpm for 10 min, 100 μL of supernatant was added to 5 mL of sterile McIlvaine buffer solution (pH 5) containing 1 μg mL^−1^ of PAT, and they were incubated at 35°C with shaking at 150 rpm for 48 h. Next, PAT was extracted from the samples and the amount of PAT was analyzed as described above. For induction of PAT degradation enzymes by PAT, the yeast cells were cultured in YPD containing 1 μg mL^−1^ of PAT at 28°C with shaking at 150 rpm overnight. Then, PAT degradation analysis followed the same procedure as described above for PAT removal test using yeast spheroplast except the use of sterile McIlvaine buffer solution (pH 4) containing 5 μg mL^−1^ of PAT instead of 1 μg mL^−1^ of PAT.

### 2.9 LC/MS/MS Analysis

PAT degradation products were identified using liquid chromatography‐tandem mass spectrometry (LC/MS/MS). The LC/MS/MS analysis was performed using an Agilent 1290 Infinity UHPLC (Santa Clara, CA, USA), which was coupled to an Agilent 6545XT LC/Quadrupole Time‐of‐Flight (Q‐TOF) LC/MS equipped with a dual‐spray Agilent Jet Stream electrospray ionization source. Separation was carried out on an Agilent ZORBOX Eclipse Plus C18 column (2.1 mm × 100 mm, 1.8 μm particle size) with a mobile phase at a constant flow rate of 0.25 mL min^−1^. The mobile phase was an online mixture (solution A:solution B = 95:5 [v/v]) of solution A (0.1% formic acid containing 5 mM NH_4_ acetate) and solution B (100% methanol). Alternatively, 100% pure water was used as a solution A, whereas 100% ACN was used as a solution B. The injection volume of samples was 20 μL, while the column temperature was maintained at 30°C.

The MS spectrometer was operated in the negative electrospray ionization (ESI) mode, and the peak spectrum was obtained by the Find by Formula data‐mining algorithm. The main MS parameters were optimized and set as follows: mass range, 50–500 amu; scan rate, 1 spectra s^−1^; gas temperature, 325°C; gas flow rate, 6 L min^−1^; nebulizer gas pressure, 45 psi; sheath gas temperature, 350°C; sheath gas flow rate, 11 L min^−1^; capillary voltage, 3500 V; skimmer, 65 V; and octupole radiofrequency voltage, 750 V. Data processing were carried out using Agilent MassHinter Qualitative Analysis Software, rev. 10.0 (Santa Clara, CA, USA).

### 2.10 Statistical Analyses

Data were statistically analyzed by a one‐way analysis of variance (ANOVA), followed by Duncan's post hoc test, and expressed as the mean ± SD using SigmaStat software (Jandel Corporation, San Rafael, CA). A *p* value < 0.05 was considered statistically different.

## 3 Results and Discussion

### 3.1 Validation of the Analytical Method for Determination of PAT Using HPLC‐UVD

**Figure A1:**
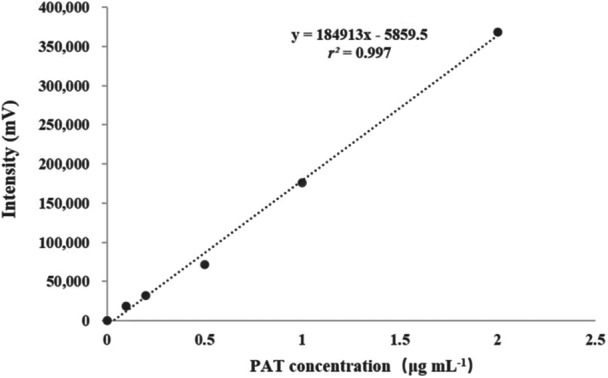
Calibration curve of PAT standard solutions for quantification of PAT in McIlvaine buffer solution (pH 4). A series of PAT standard solutions were prepared in the range of 0.1, 0.2, 0.5, 1, and 2 μg mL^−1^. Each standard solution was injected into HPLC‐UVD in triplicate.

The analytical method for determination of PAT using HPLC‐UVD was validated for analytical parameters such as linearity, accuracy, precision, and sensitivity. The linearity of the calibration curve using five levels of PAT standard solutions was determined by linear regression analysis. The curve showed *r* ^*2*^ value of 0.997 (Appendix Figure A1). Therefore, it was concluded that the calibration curve was linear in the range of 0–2 μg mL^−1^ PAT.

The accuracy of the method was evaluated by the recovery of PAT extracted from McIlvaine buffer (citric acid‐Na_2_HPO_4_, pH 4) solution spiked with three levels of PAT standard solutions (0.2, 0.5, and 1 μg mL^−1^). The recovery for PAT in McIlvaine buffer (citric acid‐Na_2_HPO_4_, pH 4) solution was in the range of 89.18%–89.99% (Table 1). The recovery rates in all of the samples were higher than 75%, which is in good agreement with the recovery rates (75%–105%) recommended by *Official Journal of the European Union* (European Commission [EC] 2006). Hence, we concluded that the analytical method had good recoveries of PAT in McIlvaine buffer solution (pH 4).

**Table 1:** Recovery and within‐day precision of PAT in McIlvaine buffer (pH 4) solution.

PAT level (μg mL^−1^)	Recovery (%)	RSDr^a^ (%)
0.2	89.18 ± 2.54	2.85
0.5	89.99 ± 3.03	3.37
1.0	89.93 ± 1.18	1.32

^a^ RSDr indicates relative standard deviation calculated from results generated under repeatability conditions.

Recovery and *RSDr* were measured in triplicate and are expressed as the mean ± standard deviation (SD).

The precision of the HPLC method was assessed by repeatability (within‐day precision). The *RSDr* obtained from extraction of PAT in McIlvaine buffer solution (pH 4) was in the range of 1.32%–3.37% (Table 1). They were consistent with the reference level (≤ 15%) recommended by *Official Journal of the European Union* (European Commission [EC] 2006). Thus, it was concluded that the analytical method showed good precision in determination of PAT in McIlvaine buffer solution (pH 4).

**Figure 1:**
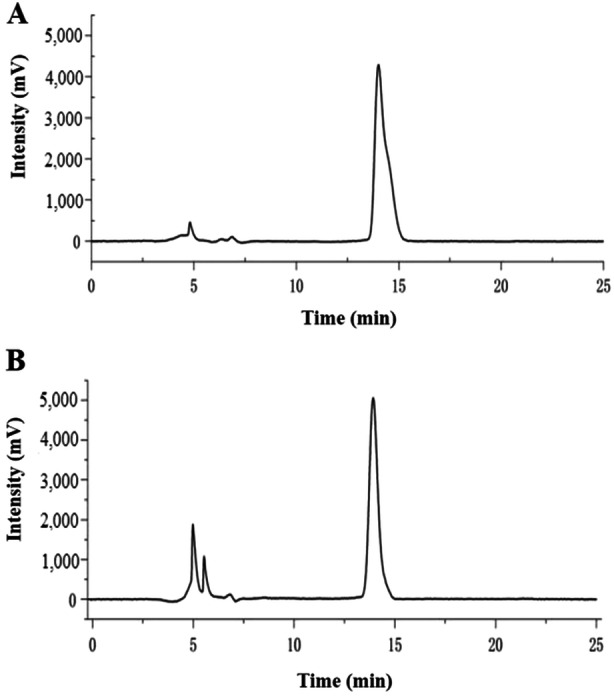
Chromatograms of PAT using HPLC‐UVD. Chromatograms of (A) PAT standard solution (1 μg mL^−1^) and of (B) PAT extracted from McIlvaine buffer solution (pH 4). The retention time (RT) of the PAT peak was 13.9 min.

The sensitivity of the HPLC‐UVD method was determined by LOD and LOQ. The LOD and LOQ for PAT in McIlvaine buffer solution (pH 4) were 0.03 and 0.11 μg mL^−1^, respectively. Since they were as low as those for detection of trace amounts of PAT, we concluded that the method was highly sensitive for determination of PAT in McIlvaine buffer solution (pH 4). Figure 1 shows the representative chromatograms of PAT in standard solution and after extraction from McIlvaine buffer solution (pH 4). As shown in Figure 1, the PAT peak at 13.9 min was well separated from the interfering peaks at 5 and 5.5 min.

### 3.2 Isolation of Yeast Strains From Peaches and Apples, and Identification of the Yeast Strains

**Figure 2:**
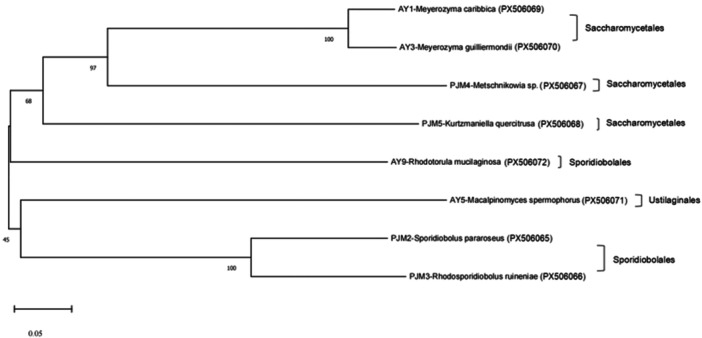
Phylogenetic relationship based on sequences of ITS1‐5.8S rDNA‐ITS2 region from yeasts isolated from peaches and apples. The number in parentheses indicates GenBank accession number. The phylogenetic tree of 8 yeasts isolated from peaches and apples was constructed using the neighbor‐joining (NJ) method.

A total of 6 and 10 yeasts were isolated from peaches and apples, respectively. Genetic identification based on ITS1‐5.8S rDNA‐ITS2 region on yeast rDNA was performed for all 16 isolated yeasts. The ITS plus 5.8S rDNA region was successfully amplified by PCR using yeast genomic DNA from all 16 yeast strains. BLASTn‐based analysis showed that sequence similarity of the yeast isolates to database in NCBI ranged from 93.45% to 99.83% (Table 2). The results exhibited that four types of yeast strains were isolated from peaches and apples, respectively, and that the yeast strains, which were isolated from peaches, were not found in those from apples and vice versa (Table 2). Of the six yeast strains isolated from peaches, *R. ruineniae* was the most predominant species (3 CFU and 50%), whereas *Sporidiobolus pararoseus*, *Metschnikowia* sp., and *Kurtzmaniella quercitrusa* were the rest of the strains (1 CFU and 16.7% for each). In contrast, of the 10 yeast strains isolated from apples, *M. guilliermondii* was the most prevalent species (5 CFU and 50%), followed by *M. caribbica* (2 CFU, 20%) and *Rhodotorula mucilaginosa* (*R. mucilaginosa*; 2 CFU, 20%), and *Macalpinomyces spermophorus* (1 CFU, 10%). When we further categorized the yeast strains, those from peaches belonged to two orders (Sporidiobolales and Saccharomycetales), whereas those from apples belonged to three orders (Saccharomycetales, Sporidiobolales, and Ustilaginales). These yeast strains, isolated from peaches and apples, differ slightly from those in previous studies (Janisiewicz et al. 2014; J. Wei et al. 2017; S. Zhu et al. 2023). One study reported that *Aureobasidium pullulans* was the most predominant species, followed by *Hanseniaspora uvarum*, *Rhodotorula glutinis*, *Cryptococcus flavescens*, and *M. guilliermondii* among yeast isolated from apples at 43 different sites in China (J. Wei et al. 2017). In particular, their study showed that geographical location was associated with the diversity of yeast genera isolated from apples. Also, Janisiewicz and co‐workers documented that *Rhodotorula* sp., *Sporidiobolus* sp., and *Aureobasidium* sp. constituted approximately 80% of all yeast isolated from plum (cultivars President and Stanley) in the United States, while Zhu and collaborators described that *Aureobasidium* sp., *Hanseniaspora* sp., *Metschnikowia* sp., *Saccharomyces* sp., *Pichia* sp., *Rhodotorula* sp., *Sporobolomyces* sp. were dominant species isolated from fruits, leaves, and flowers of peach trees (*Amygdalus persica* L. Compressa) (Janisiewicz et al. 2014; S. Zhu et al. 2023). In addition, one study from Slovenia reported that natural yeast flora on grape berries depends on different grape vine varieties as well as different geographical locations (Raspor et al. 2006). Thus, the discrepancy in our study may have been attributed to differences in geographical location and/or cultivars of peaches and apples. The phylogenetic tree based on ITS plus 5.8S rDNA sequences from eight isolates is shown in Figure 2.

**Table 2:** Identification of yeast strains isolated from peaches and apples using BLASTn‐based analysis.

Type of fruit	Sample ID	Scientific name (BLASTn accession no.^a^)	Sequence similarity (%)
Peach	PJM1	*Rhodosporidiobolus ruineniae* (HQ670680.1)	96.34
Peach	PJM2	*Sporidiobolus pararoseus* (MN340037.1)	98.75
Peach	PJM3	*Rhodosporidiobolus ruineniae* (HQ670680.1)	99.17
Peach	PJM4	*Metschnikowia* sp. (AB998445.1)	96.94
Peach	PJM5	*Kurtzmaniella quercitrusa* (MH979687.1)	96.47
Peach	PJM6	*Rhodosporidiobolus ruineniae* (HQ670680.1)	93.45
Apple	A‐Y1	*Meyerozyma caribica* (MT508806.1)	99.68
Apple	A‐Y2	*Meyerozyma guilliermondii* (ON142338.1)	99.24
Apple	A‐Y3	*Meyerozyma guilliermondii* (ON142338.1)	99.83
Apple	A‐Y4	*Rhodotorula mucilaginosa* (KF728812.1)	97.34
Apple	A‐Y5	*Macalpinomyces spermophorus* (LC214955.1)	96.55
Apple	A‐Y6	*Meyerozyma guilliermondii* (ON142338.1)	94.63
Apple	A‐Y7	*Meyerozyma caribica* (MT508806.1)	98.97
Apple	A‐Y8	*Meyerozyma guilliermondii* (ON142338.1)	94.63
Apple	A‐Y9	*Rhodotorula mucilaginosa* (KF728812.1)	99.44
Apple	A‐Y10	*Meyerozyma guilliermondii* (ON142338.1)	98.24

^a^ BLASTn was run using ITS1‐5.8S rDNA‐ITS2 sequences.

### 3.3 Selection of PAT Removal Yeast Strains

In order to compare PAT removal rates by the isolated yeast strains, a total of eight yeast strains from peaches and apples were cultured in YPD (pH 4) containing 1 μg mL^−1^ of PAT at 28°C for 48 h after selection of one yeast stain from several same strains. The PAT removal rates from the eight yeast strains ranged from 81.16% to 92.4% (Table 3). *R. ruineniae*, which was isolated from a peach, produced the highest PAT removal rate (92.4%), and it was followed by *M. guilliermondii* (91.69%), which was isolated from an apple. However, the difference between their rates was not statistically significant (*p* < 0.05). The control without yeast strains also showed 12.86% of PAT removal rates, which were in line with other studies (15.4% after incubation at 28°C for 24 h in Dong and collaborators' study, and 5%–7.7% at 25°C for 48 h in Reddy and co‐workers' study) (Dong et al. 2015; Reddy et al. 2011). In addition, biomass (OD at 600 nm) of the eight yeast strains was in the range of 0.15–0.42 (Table 3). *M. guilliermondii* showed the highest cell mass (0.42), indicating that it had strong tolerance to PAT, but *R. ruineniae* showed the medium level of cell mass (0.29). Thus, we selected *R. ruineniae*, which belongs to Sporidiobolales (Basidiomycota), and *M. guilliermondii*, which belongs to Saccharomycetales (Ascomycota), for further study.

**Table 3:** Biomass and PAT removal rates of yeast strains after culture in YPD (pH 4) at 28°C with shaking at 150 rpm for 48 h.

Yeast strain (Sample ID, GenBank accession number)	Biomass (OD_600nm_)	PAT removal rate (%)
*Sporidiobolus pararoseus* (PJM2, PX506065)	0.26 ± 0.01*	90.59 ± 0.14*
*Rhodosporidiobolus ruineniae* (PJM3, PX506066)	0.29 ± 0.01*, ****	92.40 ± 0.32**
*Metschnikowia* sp. (PJM4, PX506067)	0.15 ± 0.01**	89.90 ± 0.40*
*Kurtzmaniella quercitrusa* (PJM5, PX506068)	0.28 ± 0.01*, ****	87.58 ± 0.98***
*Meyerozyma caribbica* (A‐Y1, PX506069)	0.38 ± 0.02***	87.08 ± 0.90***
*Meyerozyma guilliermondii* (A‐Y3, PX506070)	0.42 ± 0.01***	91.69 ± 0.37**
*Macalpinomyces spermophorus* (A‐Y5, PX506071)	0.30 ± 0.02*, ****	81.16 ± 1.25****
*Rhodotorula mucilaginosa* (A‐Y9, PX506072)	0.35 ± 0.05***, ****	85.85 ± 1.02***
Control (sterile DW without yeast)	—	12.86 ± 3.23*****

*Note:* The different number of the asterisk in the same column indicates statistically significant difference between data (*p* < 0.05 analyzed by ANOVA).

Data were measured in triplicate and are expressed as the mean ± standard deviation (SD).

### 3.4 PAT Removal Rates by *R. ruineniae* or *M. guilliermondii* Under Different Conditions

**Figure 3:**
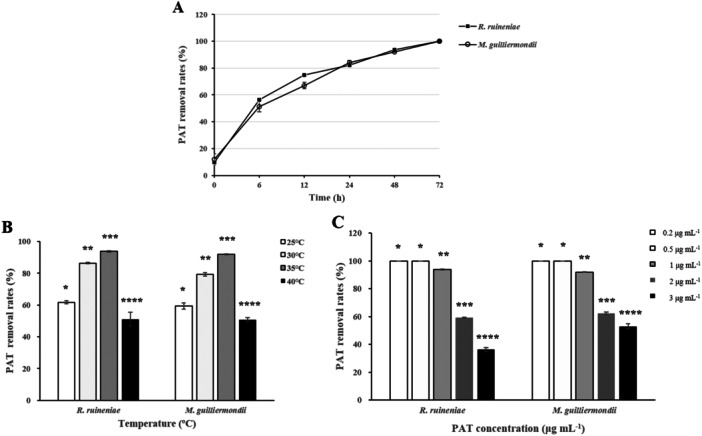
Effects of different environmental factors on PAT removal rates by *R. ruineniae* or *M. guilliermondii* in McIlvaine buffer solution (pH 4) containing PAT with shaking at 150 rpm. (A) Effect of incubation time on PAT removal rates in the buffer containing 1 μg mL^‐1^ of PAT at 35°C, (B) effect of incubation temperature on PAT removal rates in the buffer containing 1 μg mL^‐1^ of PAT after 48 h, and (C) effect of initial PAT concentration on PAT removal rates in the buffer containing different levels of PAT after 48 h at 35°C. The PAT removal rates were measured in triplicate. Data are expressed as the mean ± standard deviation (SD). The different number of the asterisk in the same group indicates statistically significant differences (*p* < 0.05).

Since several parameters such as incubation time and temperature, and initial PAT concentration affect PAT removal rates, we investigated the effects of the different environmental factors on PAT removal rates by *R. ruineniae* and *M. guilliermondii*. When the two yeast strains were incubated in 5 mL of McIlvaine buffer solution (pH 4) containing 1 μg mL^−1^ of PAT over 72 h, PAT removal rates were similar between the yeast strains (Figure 3A). The rates in both strains reached the lower level than the regulatory limit (50 µg L^−1^) at 60 h and PAT was completely degraded (100%) at 72 h. To analyze the effect of incubation temperature on PAT removal rates by the two yeast strains, we incubated the yeast strains in 5 mL of McIlvaine buffer solution (pH 4) containing 1 μg mL^−1^ of PAT at four different temperature conditions (25°C, 30°C, 35°C, and 40°C) for 48 h. The PAT removal rates showed a similar pattern between *R. ruineniae* and *M. guilliermondii*. They were highest (93.8% in *R. ruineniae* and 91.98% in *M. guilliermondii*) at 35°C, followed by 30°C, 25°C, and 40°C (Figure 3B). These results were in agreement with those by Dong and co‐workers' study, in which a marine yeast strain *Kodameae ohmeri* (*K. ohmeri*) produced the highest PAT degradation rate (93%) at 35°C, followed by 30°C, 25°C, and 40°C after 20 h incubation (Dong et al. 2015). Another study reported that *R. mucilaginosa* had the highest PAT degradation rate (90%) after 21 h incubation, which is similar to our results (X. Li, Tang, et al. 2019). To assess the effect of initial PAT concentrations on PAT removal rates by the two yeast strains, they were incubated in 5 mL of McIlvaine buffer solution (pH 4) containing 0.2–3 μg mL^−1^ of PAT at 35°C for 48 h. With an increase in initial PAT concentrations, PAT removal rates in both yeast strains gradually decreased (Figure 3C). The rates in *R. ruineniae* and *M. guilliermondii* were 100% when 0.2 or 0.5 μg mL^−1^ of the initial PAT concentration was used, while they were 36.22% and 52.9%, respectively, when 3 μg mL^−1^ of the initial PAT concentration was used. Thus, our data showed that both yeast strains reduced the levels of PAT in buffer solutions in an incubation time and temperature‐ and initial PAT concentration‐dependent manner.

### 3.5 Mechanism of PAT Removal by *R. ruineniae* or *M. guilliermondii*

**Figure 4:**
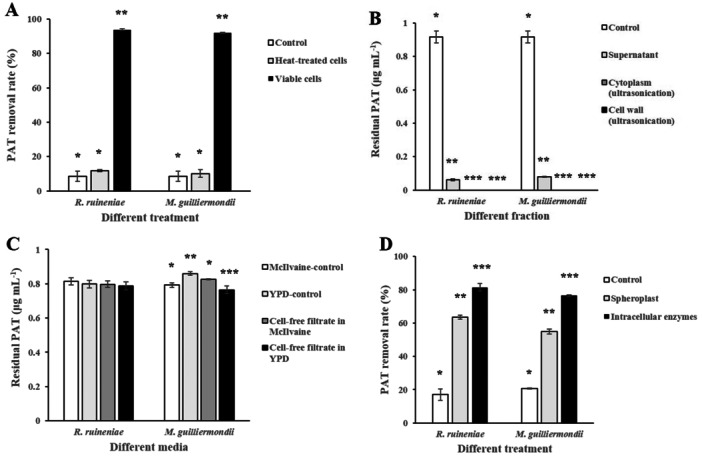
PAT removal rates by different cell treatment or different cell fractions of *R. ruineniae* or *M. guilliermondii* after 48 h at 35°C with shaking at 150 rpm. (A) Heat‐treated or viable yeast cells, (B) yeast cell walls or cytoplasms isolated after cell disruption by ultrasonication, (C) yeast cell‐free filtrates in McIlvaine buffer solution (pH 4) or YPD (pH 4) after culture in either medium, and (D) yeast spheroplast and intracellular enzymes. The PAT removal rates were measured in triplicate. Data are expressed as the mean ± standard deviation (SD). The different number of the asterisk in the same group indicates statistically significant differences (*p* < 0.05).

Some previous studies reported that heat‐inactivated yeast strains using *S. cerevisiae* or *Rhodosporidium paludigenum* (*R. paludigenum*) had significant PAT removal rates, which were comparable to those by viable yeast cells (Chen et al. 2017; Guo et al. 2012; Yue et al. 2011; R. Zhu, Yu, et al. 2015). Thus, after autoclaving live yeast cells at 121°C for 15 min, we used the heat‐treated yeast cells (dead yeast cells) to compare their PAT removal rates with those by viable yeast cells. The viable yeast cells of *R. ruineniae* or *M. guilliermondii* showed 93.8% and 91.98% of PAT degradation rates, respectively, after 48 h incubation, whereas the heat‐treated yeast cells showed 11.68% and 10.13% of PAT degradation rates, which were not different from that of a control without yeast strains (8.4%) (Figure 4A). These results are consistent with those from several previous studies (Fu et al. 2021; Zheng et al. 2016). One previous study documented that heat‐inactivated *M. guilliermondii* did not reduce much PAT content (10% reduction) after 48 h incubation (Fu et al. 2021). Another study described that PAT removal rate by heat‐killed *P. caribbica* after 36 h culture was similar to that by a control without the yeast strain (30% reduction) (Zheng et al. 2016). However, our data are different from those of the other previous studies (Chen et al. 2017; Guo et al. 2012; Yue et al. 2011; R. Zhu, Yu, et al. 2015). Guo and co‐workers reported that there was no significant difference between PAT removal rates of heat‐inactivated yeast cells (51.71%) and viable cells (53.28%) after 24 h incubation (Guo et al. 2012). Other studies also showed 40%–60% of PAT degradation rates using heat‐inactivated yeast cells after 24 or 48 h incubation (Chen et al. 2017; Yue et al. 2011; R. Zhu, Yu, et al. 2015). This discrepancy may have resulted from the use of different yeast strains (*S. cereviaise*, *R. paludigenum*, and *C. guilliermondii*). Also, in Guo and co‐workers' study they described that most of the PAT removal was due to adsorption onto cell walls of activated or inactivated *S. cereviaise* (Guo et al. 2012). In our study, PAT removal by viable cells of *R. ruineniae* or *M. guilliermondii* could have resulted from physical adsorption of PAT onto yeast cell walls or degradation of PAT by the yeast strains. Thus, in order to isolate yeast cell walls, we disrupted yeast cells by ultrasonication or grinding under liquid N_2_ and isolated the yeast cell walls. After PAT extraction, HPLC‐UVD analysis showed that PAT was not detected from the cell walls and cytoplasms of both yeast strains, which were prepared by either method (Figures 4B and Appendix B1). It is in agreement with several other studies (Dong et al. 2015; Reddy et al. 2011). One study showed that no PAT was detected in the cell wall and cytoplasm of *K. ohmeri*, which were isolated by grinding under liquid N_2_ (Dong et al. 2015). Another study described that no detectable level of PAT was observed in the cell wall of *Metschnikowia pulcherrima*, which was isolated by ultrasonication (Reddy et al. 2011). Our data suggested that PAT was degraded by the yeast strains instead of binding of PAT onto yeast cell walls. Hence, we first investigated PAT removal rates by yeast cell‐free filtrates. However, the yeast cell‐free filtrates from yeast culture in YPD (pH 4) or McIlvaine buffer solution (pH 4) did not increase PAT removal rates in the media or buffer solution (pH 4) compared to the controls without cell‐free filtrates after 48 h incubation (Figure 4C). These results are consistent with several previous studies, which showed that extracellular filtrates of *M. guilliermondii* or *R. paludigenum* culture did not show any difference from PAT removal rate (approximately 10%) by a control without the yeast strain after 48 h culture and that cell‐free filtrates of *C. guilliermondii* produced the similar PAT removal rate (approximately 20%) to that by a control after 24 h incubation (Chen et al. 2017; Fu et al. 2021; R. Zhu, Feussner, et al. 2015). However, one study reported different results (Zheng et al. 2016). Zheng and collaborators described that cell‐free filtrates of *P. caribbica* reduced PAT level significantly (45% reduction) compared to a control without the yeast strain (25% reduction) after 24 h incubation (Zheng et al. 2016). Again, this result may have come from the use of different yeast strains. Nevertheless, our results indicated that the PAT degradation is an enzyme‐mediated mechanism inside yeast cells. Because our data suggested that PAT removal by both yeast strains was not due to either binding onto yeast cell walls or degradation by extracellular enzymes in cell‐free filtrates, we next analyzed PAT removal rates using yeast spheroplast, in which cell walls are deficient. In contrast to the results from the use of yeast cell walls or cell‐free filtrates, PAT removal rates by yeast spheroplast were 63.49% in *R. ruineniae* and 54.95% in *M. guilliermondii* (Figure 4D). It indicates that intracellular enzymes in yeast strains degraded PAT. Thus, to confirm PAT degradation by intracellular enzymes, we analyzed PAT removal rates using intracellular enzymes isolated from the yeast strains after cell disruption by grinding under liquid N_2_. The PAT removal rates using intracellular enzymes were approximately 1.3‐fold higher than those using spheroplast from both yeast strains (Figure 4D). These results are in line with those from some previous studies (Chen et al. 2017; Fu et al. 2021; M. Li et al. 2018). One previous study described that the PAT degradation process occurred inside *C. guilliermondii* cells (Chen et al. 2017). Another study showed that the protoplasts, in which cell walls are completely removed, of *M. guilliermondii* produced lower PAT removal rates than its viable cells after 48 or 60 h incubation (Fu et al. 2021). Moreover, Li and co‐workers reported that both intracellular and extracellular enzymes from *S. cerevisiae* CITCC 93161 were responsible for PAT degradation (M. Li et al. 2018).

**Figure 5:**
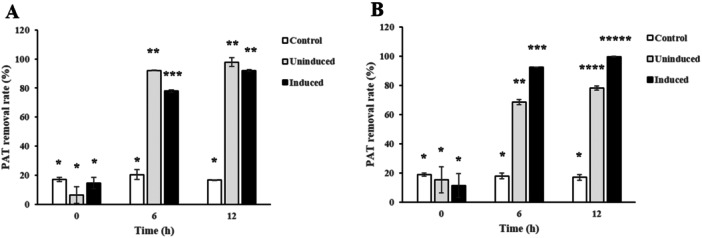
PAT removal rates by spheroplast of *R. ruineniae* or *M. guilliermondii* after 48 h at 35°C with shaking at 150 rpm when the yeast strains were cultured in YPD supplemented with or without 1 μg mL^‐1^ of PAT. (A) Spheroplast of *R. ruineniae* and (B) spheroplast of *M. guilliermondii*. The PAT removal rates were measured in triplicate. Data are expressed as the mean ± standard deviation (SD). The different number of the asterisk in the same group indicates statistically significant differences (*p* < 0.05).

Several previous studies documented that PAT degradation ability using yeast strains was induced by addition of PAT to culture media (Fu et al. 2021; Ianiri et al. 2017; Zheng et al. 2016; R. Zhu, Feussner, et al. 2015). Hence, we cultured *R. ruineniae* or *M. guilliermondii* in YPD containing 5 μg mL^−1^ of PAT and prepared spheroplasts of the yeast strains. Then, PAT degradation test was performed using the spheroplast of each strain. HPLC analysis showed that PAT degradation enzymes were not induced in *R. ruineniae*, whereas they were induced in *M. guilliermondii* (Figure 5A,B). The result on the induced PAT degradation ability in *M. guilliermondii* is consistent with those from previous studies (Fu et al. 2021; Ianiri et al. 2017; M. Li et al. 2018; Zheng et al. 2016; Zhu, Feussner, et al. 2015). In one study, Fu and collaborators described that the addition of PAT to culture media enhanced the PAT degradation ability of intracellular enzymes from *M. guilliermondii* (Fu et al. 2021). Other studies also reported that PAT‐degrading activities of intracellular enzymes from *R. paludigenum*, *P. caribbica*, or *Sporobolomyces* sp. were induced by addition of PAT to culture media (Ianiri et al. 2017; Zheng et al. 2016; R. Zhu, Feussner, et al. 2015). However, one previous study showed that the PAT degradation ability of intracellular enzymes from *S. cerevisiae* was not induced by culture media supplemented with PAT (M. Li et al. 2018), which is similar to that by *R. ruineniae* in our results.

### 3.6 PAT Degradation Products by *R. ruineniae* or *M. guilliermondii*

**Figure 6:**
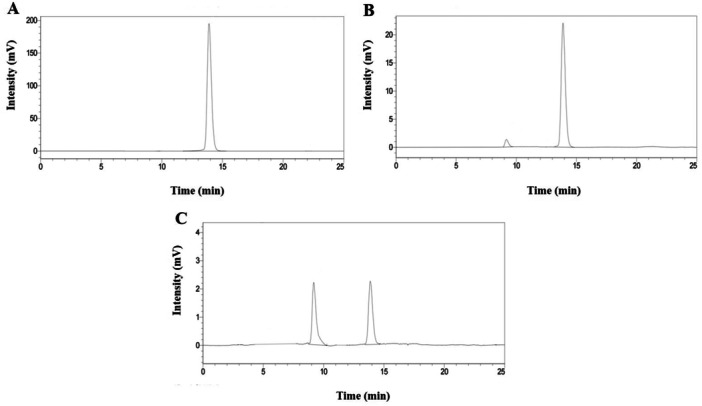
Time‐course HPLC chromatograms of McIlvaine buffer solution (pH 4) containing 5 μg mL^‐1^ of PAT when treated with spheroplast of *R. ruineniae* at 35°C with shaking at 150 rpm. (A) 0 h incubation, (B) 12 h incubation, and (C) 24 h incubation. The retention time (RT) of the PAT peak was 13.9 min, whereas that of E‐ascladiol peak was 9.2 min.

**Figure C1:**
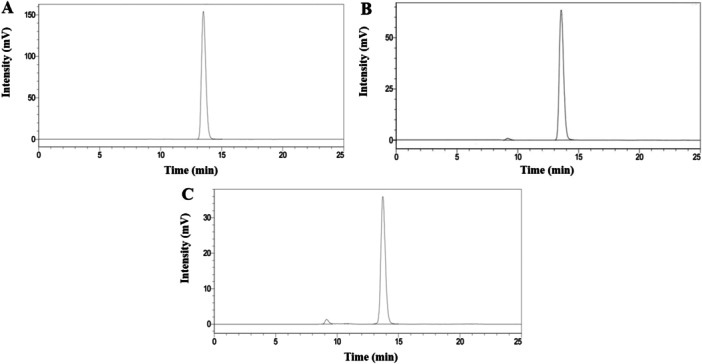
Time‐course HPLC chromatograms of McIlvaine buffer solution (pH 4) containing 5 μg mL^−1^ of PAT when treated with spheroplast of *M. guilliermondii* at 35°C with shaking at 150 rpm. (A) 0 h incubation, (B) 12 h incubation, and (C) 24 h incubation. The retention time (RT) of the PAT peak was 13.6 min, whereas that of (E)‐ascladiol peak was 9.2 min.

In order to identify PAT degradation products after treatment with the spheroplasts of *R. ruineniae* or *M. guilliermondii*, time‐course experiments were performed. After 12 h incubation with the spheroplasts of *R. ruineniae*, a new peak appeared at 9.2 min, while the PAT peak at 13.9 min decreased (Figure 6A,B). As the incubation time increased to 24 h, the PAT peak decreased further, but the new peak became higher (Figure 6C). Moreover, treatment with the spheroplast of *M. guilliermondii* showed a similar pattern in HPLC chromatograms to that of *R. ruineniae* (Appendix Figure C1).

**Figure 7:**
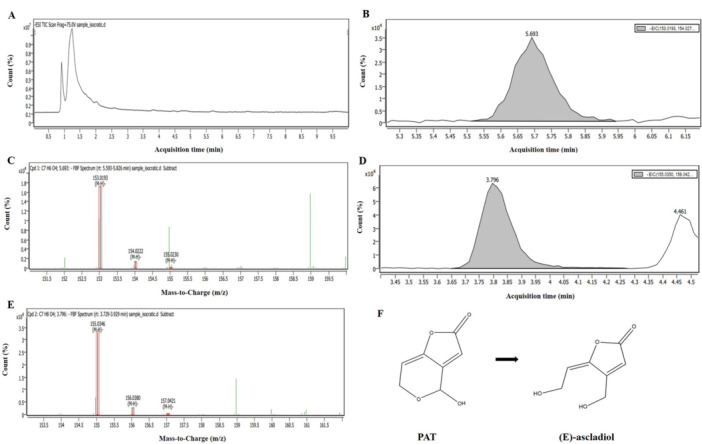
Total and extracted ion chromatograms (TIC and EIC) and MS spectra of PAT and (E)‐ascladiol in McIlvaine buffer solution (pH 4) containing 5 μg mL^−1^ of PAT when treated with spheroplast of *R. ruineniae* for 24 h at 35°C with shaking at 150 rpm. (A) TIC, (B) PAT peak (RT = 4.372 min) in EIC, (C) MS spectrum for PAT, (D) (E)‐ascladiol peak (RT = 2.675 min) in EIC, (E) MS spectrum for (E)‐ascladiol, and (F) conversion of PAT to (E)‐ascladiol.

**Figure D1:**
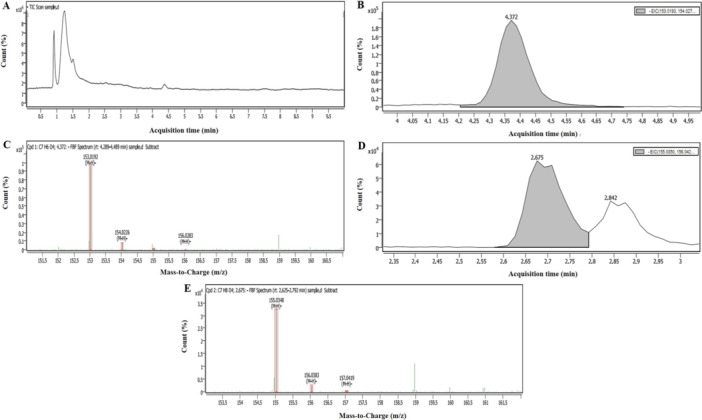
Total and extracted ion chromatograms (TIC and EIC) and MS spectra of PAT and (E)‐ascladiol in McIlvaine buffer solution (pH 4) containing 5 μg mL^−1^ of PAT when treated with spheroplast of *M. guilliermondii* for 36 h at 35°C with shaking at 150 rpm. (A) TIC, (B) PAT peak (RT = 4.372 min) in EIC, (C) MS spectrum for PAT, (D) (E)‐ascladiol peak (RT = 2.675 min) in EIC, and (E) MS spectrum for (E)‐ascladiol.

LC/MS/MS analysis detected not only [M‐H]^−^ precursor (*m*/*z* = 153.0193) at 5.693 min, which corresponds to PAT (molecular mass = 154.0266, C_7_H_6_O_4_) but also [M‐H]^−^ precursor (*m*/*z* = 155.0346) at 3.796 min in the same samples as those analyzed by HPLC‐UVD after they were treated with spheroplast of *R. ruineniae* (Figure 7A–E). It was revealed that the signal associated with the *m*/*z* 155.0346 at 3.796 min corresponds to (E)‐ or (Z)‐ascladiol with the formula C_7_H_8_O_4_ ([M‐H]^−^ precursor, theoretical *m*/*z* = 155.0352). Also, the LC/MS/MS analysis exhibited a similar pattern in *M. guilliermondii* to *R. ruineniae*. It showed [M‐H]^−^ precursor (*m*/*z* = 153.0192) at 4.372 min, which corresponds to PAT, and [M‐H]^−^ precursor (m/z = 155.0348) at 2.675 min, which corresponds to (E)‐ or (Z)‐ascladiol, in the same samples as those analyzed by HPLC‐UVD after they were treated with spheroplast of *M. guilliermondii* (Appendix Figure D1). In addition, the peak of (E)‐ or (Z)‐ascladiol appeared earlier than PAT peak in the HPLC chromatogram as shown in Figure 6. It is in good agreement with the results from several previous studies (Chen et al. 2017; Dong et al. 2015; M. Li et al. 2018; Moss and Long 2002; Yang et al. 2024). Some previous studies reported that *S. cerevisiae* degraded PAT to the less toxic product (E)‐ascladiol than the parent compound (M. Li et al. 2018; Moss and Long 2002; Yang et al. 2024). Other studies also documented that the major PAT degradation product is (E)‐ascladiol when *K. ohmeri* or *C. guilliermondii* was used (Chen et al. 2017; Dong et al. 2015). In particular, in Chen and co‐workers' study, the retention time (RT) of (E)‐ascladiol and PAT peaks was 6.0 and 10.2 min, respectively, when the mobile phase was 10% ACN in DW with a flow rate of 1 mL min^−1^ (Chen et al. 2017). Another previous study showed that the peak of (E)‐ascladiol (RT = 5.85 min) appeared first, followed by those of (Z)‐ascladiol (RT = 9.26 min) and PAT (RT = 9.92 min) when they were analyzed under the similar HPLC condition to that in Chen and co‐workers' study (Dong et al. 2015). Thus, in our study, we assume that the PAT degradation product by *R. ruineniae* or *M. guilliermondii* is (E)‐ascladiol (Figure 7E), an immediate precursor of PAT in the biosynthetic pathway (B. Li, Chen, et al. 2019).

To date, two major products have been reported in degradation of PAT by yeast strains: (E)‐ or (Z)‐ascladiol (two different isomers) and desoxypatulinic acid (McCormick 2013). In three previous studies, the authors showed that *Rhodosporidium kratochvilovae* (*R. kratochvilovae*), *R. paludigenum*, or *R. mucilaginosa* converted PAT to another less toxic product, desoxypatulinic acid, whose peak appeared later than PAT peak in the HPLC chromatogram (Castoria et al. 2011; X. Li, Tang, et al. 2019; R. Zhu, Feussner, et al. 2015). Furthermore, two previous studies documented that a yeast strain converted PAT into two different degradation products (ascladiol and desoxypatulinic acid) (Ianiri et al. 2013; Zhang et al. 2025). One study described that *K. marxianus* XZ1 was able to degrade PAT to produce both types of the PAT degradation products (Zhang et al. 2025). Ianiri and collaborators also showed the similar results to the study, in which *Sporobolomyces* sp. strain IAM 13481 converted PAT to form both types of the PAT breakdown products (Ianiri et al. 2013). In their study, the peak of (E)‐ or (Z)‐ascladiol appeared earlier than PAT peak in the HPLC chromatogram, while the peak of desoxypatulinic acid appeared later than the PAT peak in the chromatogram when the mobile phase was 5% methanol in acidified DW containing 1% acetic acid with a flow rate of 1 mL min^−1^.

PAT contamination on pome fruits such as apples and peaches is a serious health risk worldwide (Mahato et al. 2021; Moake et al. 2005). Development of PAT biodegradation strategies using microorganisms has received considerable attention. In this study, we isolated *R. ruineniae* and *M. guilliermondii* as PAT‐degrading yeast strains from a peach and an apple, respectively, and investigated the mechanism involved in PAT degradation and the major biodegradation product. Our data showed that several parameters such as incubation time and temperature, and also initial PAT concentration had significant effects on PAT degradation rate in both yeast strains. Both yeast strains were able to degrade 1 μg mL^−1^ of PAT to below the regulatory limit (50 µg L^−1^) at 60 h when they were incubated at 35°C. In addition, PAT was not detected from yeast cell walls of *R. ruineniae* or *M. guilliermondii*, whereas it was degraded by spheroplast or intracellular enzyme fractions of the yeast strains. It indicates that PAT was not adsorbed onto the yeast cell walls, but PAT degradation occurred by intracellular enzymes of the yeast strains. Also, PAT degradation ability of intracellular enzymes from *M. guilliermondii* was induced by addition of PAT to culture media. Furthermore, LC/MS/MS analysis showed that (E)‐ascladiol is a major PAT biodegradation product by spheroplast of *R. ruineniae* or *M. guilliermondii*.

Interestingly, Iarniri and co‐workers described that the production of desoxypatulinic acid has been found in the Pucciniomycotina of basidiomycetes to which red yeasts such as *Rhodosporidium* sp. and *Rhodotorula* sp. belong, while the ascomycete *S. cerevisiae*, *P. caribbica*, or *C. guilliermondii* converts PAT to ascladiol as a major degradation product (Ianiri et al. 2016, 2017). To the best of our knowledge, this is the first report on PAT biodegradation using *R. ruineniae* and production of (E)‐ascladiol by the red yeast.

PAT contamination of apples and apple‐based products is a worldwide issue. High PAT levels, which exceed the maximum regulatory limit (50 µg kg^−1^), were detected in apples and apple juice from China, Spain, and Pakistan (Zhong et al. 2018). Since apples are frequently contaminated with PAT, which is produced by infected fungi such as *Penicillium expansum*, during postharvest storage, crude protein extracts of *R. ruineniae* and *M. guilliermondii*, which were isolated as PAT‐degrading yeast strains in this study, could be utilized for PAT decontamination of the stored fruits by spraying them onto the fruits in the future.

## 4 Conclusion

In summary, our data demonstrated that *R. ruineniae* and *M. guilliermondii* isolated from a peach and an apple, respectively, were able to degrade PAT and that their spheroplast formed (E)‐ascladiol as the sole breakdown product. In addition, it showed that PAT degradation rates by each yeast strain were significantly influenced by incubation time and temperature, and initial PAT concentration. Future studies will reveal the enzymes involved in PAT biodegradation and the detailed mechanisms for conversion of PAT to (E)‐ascladiol by the yeast strains. Furthermore, the potential application of the crude protein extracts from *R. ruineniae* and *M. guilliermondii* to apples could be helpful for PAT decontamination of the fruits.

## Author Contributions

**Yidan Ji:** investigation (lead), formal analysis (lead), writing – review and editing (equal). **Sung‐Yong Hong:** conceptualization (equal), formal analysis (lead), writing – original draft (lead), writing – review and editing (equal), supervision (equal). **Jinhuan Qu:** investigation (supporting), formal analysis (supporting), writing – review and editing (equal). **Qing Chu:** investigation (supporting), formal analysis (supporting), writing – review and editing (equal). **Shuxian Ma:** investigation (supporting), formal analysis (supporting), writing – review and editing (equal). **Ae‐Son Om:** conceptualization (equal), writing – review and editing (equal), supervision (equal), funding acquisition.

## Funding

This study was supported by the research program (Development of data application technology for postharvest management of the agricultural and livestock products; Project No. RS‐2022‐RD010281) from Rural Development Administration, Korea.

## Ethics Statement

The authors have nothing to report.

## Conflicts of Interest

The authors have nothing to report.

## Figures and Tables

**Figure B1:**
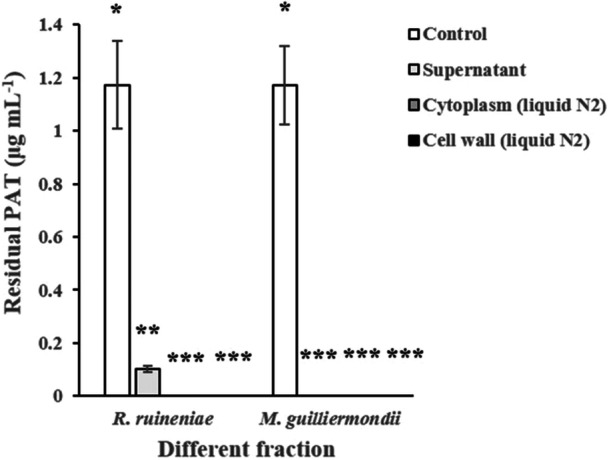
PAT removal rates by different cell fractions of *R. ruineniae* or *M. guilliermondii* after 48 h at 35°C with shaking at 150 rpm when the cells were disrupted under liquid N_2_. The PAT removal rates were measured in triplicate. Data are expressed as the mean ± standard deviation. The different number of the asterisk in the same group indicates statistically significant differences (*p* < 0.05).

## Data Availability

The data that support the findings of this study are available in the supporting material of this article.

## Appendix A

**Calibration curve of PAT standard solutions**

## Appendix B

**PAT removal rates using different cell fractions of** ***R. ruineniae*** **or** ***M. guilliermondii*** **obtained from cell disruption by liquid N**_**2**_

## Appendix C

**Time‐course HPLC chromatograms of PAT degradation using spheroplast of** ***M. guilliermondii***

## Appendix D

**TIC, EIC, and MS spectra of PAT and (E)‐ascladiol in PAT degradation using spheroplast of** ***M. guilliermondii***
